# The role of intracellular and extracellular copper compartmentalization in Alzheimer’s disease pathology and its implications for diagnosis and therapy

**DOI:** 10.3389/fnins.2025.1553064

**Published:** 2025-03-12

**Authors:** Yu-Qi Li, Shuang-Shuang Tan, Di Wu, Qian Zhang, Tao Wang, Gang Zheng

**Affiliations:** ^1^School of Public Health, Gansu University of Chinese Medicine, Lanzhou, China; ^2^School of Military Preventive Medicine and the Ministry of Education Key Lab of Hazard Assessment and Control in Special Operational Environment, Fourth Military Medical University, Xi’an, China; ^3^Research Institution, Xijing Hospital, Fourth Military Medical University, Xi’an, China; ^4^Department of Psychosomatic Medicine, Xijing Hospital, Fourth Military Medical University, Xi’an, China; ^5^Center of Clinical Aerospace Medicine and Department of Aviation Medicine, Fourth Military Medical University, Xi’an, China

**Keywords:** Alzheimer’s disease, copper homeostasis, oxidative stress, amyloid precursor protein, diagnosis

## Abstract

Copper is a trace element indispensable for cellular physiology, integral to cellular redox balance, and a constituent of enzyme active sites, thereby playing a pivotal role in cellular physiological function. Concerning the pathogenesis of Alzheimer’s disease (AD), the homeostatic balance of copper is perturbed both intracellularly and extracellularly. The copper–amyloid precursor protein (APP) complex facilitates the efflux of copper from cells, leading to intracellular copper depletion. Concurrently, extracellular copper associates with amyloid-beta (Aβ) plaques, precipitating copper-enriched Aβ deposition and augmenting reactive oxygen species (ROS) in the brain tissue, which finally culminates in oxidative brain damage. The interaction between copper and APP enhances the α-secretase pathway of APP processing while suppressing the β-secretase pathway, resulting in an increased production of soluble APP (sAPP), which contributes to neuroinflammation in the brain tissue. Utilizing the affinity of copper for Aβ plaques, the application of chelating agents to sequester copper within the brain can mitigate neurodegeneration associated with AD pathology. Furthermore, the use of metal imaging techniques to detect copper in the brain offers a potential diagnostic tool for the early identification of AD.

## 1 Introduction

Copper is a critical micronutrient indispensable for various physiological functions in humans. A healthy adult body contains approximately 110 mg of copper, primarily concentrated in the liver, cerebral cortex, circulatory system, skeletal structures, and muscle compartments. Diet is the main source of copper for the body. As a transition metal and an essential cofactor for redox enzymes, copper participates in cellular oxidation–reduction processes, mitochondrial respiration, antioxidant defense, and the synthesis of biological compounds. The absorption, distribution, and elimination of copper are tightly regulated. An increase in intracellular copper can lead to cytotoxicity and cell death, while copper deficiency can result in insufficient cellular antioxidant capacity, causing oxidative damage. Copper is also involved in angiogenesis, the formation of neural myelin sheaths, and the action of endorphins. Maintaining copper homeostasis in the body is crucial.

Copper exerts a significant influence on the initiation and advancement of Alzheimer’s disease (AD). It has been found that there are significant abnormalities in the distribution of copper in the brain tissue of AD patients. The balance of both intracellular and extracellular copper homeostasis is affected, and the imbalance of copper homeostasis in the brain exacerbates the pathogenesis of AD. Extracellular copper ions can bind directly to Aβ amyloid, and there are two copper-binding sites in the Aβ precursor protein (APP), located in the Aβ region and the N-terminal region, which promote the aggregation of Aβ monomers and the formation of more toxic Aβ fibrils and plaques. When copper(II) binds to Aβ, this binding also induces the generation of hydroxyl radicals and exacerbates oxidative stress. Intracellular copper ions can affect the hyperphosphorylation of tau proteins through a variety of mechanisms, including altering the conformation of tau proteins and activating kinases (e.g., GSK3β), which in turn leads to the formation of neurofibrillary tangles, altogether disrupting intracellular signaling pathways and exacerbating the pathological process in AD patients.

## 2 Copper metabolism and its influence on the occurrence and progression of AD

### 2.1 Regulation of copper metabolism in organ cells

Copper ions absorbed by the small intestine bind to ceruloplasmin for transport in the bloodstream, with the majority of the copper eventually being transferred to the liver, kidneys, and brain. Copper exists in the body in two forms: bound copper and free copper. Bound copper refers to copper bound to biological molecules within biological systems. This form of copper is generally more stable and less reactive than free or ionic copper, which exists as a simple metal ion in solution. The blood–brain barrier (BBB) exhibits high permeability to low-molecular-weight compounds, and studies have shown that the efficiency of free copper entering the brain is 1,000 times higher than that of bound copper (Salustri et al., 2010; Choi and Zheng, 2009). Free ionic copper is absorbed into cells through the mediation of copper transport proteins (CTR1) and divalent metal transporter 1 (DMT1), while P-type ATPase copper transporters ATP7A and ATP7B facilitate the excretion of copper from cells. Brain copper concentrations are determined by the balance between copper influx and efflux. Research indicates that elevated levels of unbound ionic copper are closely associated with the onset and progression of AD (Arnal et al., 2010). Research has indicated a correlation between the use of copper pipes and a heightened incidence of AD within certain developing countries (Brewer, 2019).

The permeability of copper into the brain is governed by the BBB, where the blood–cerebrospinal fluid (CSF) barrier serves as a key regulatory checkpoint. The choroid plexus possesses various copper transport proteins that determine the rate and direction of copper traffic between the CSF and blood, acting as the main regulatory factor for copper transport across the CSF (Zheng and Monnot, 2012). The clearance rate of copper in the CSF decreases with age. Studies have shown that the copper clearance rate in the choroid plexus of aged rats is significantly reduced by 16% compared to that of young and adult rats. CTR1 and DMT1 are two copper uptake proteins that transport copper from the extracellular matrix into choroid plexus epithelial cells. Aging significantly reduces the expression of CTR1 but does not significantly alter the expression of DMT1. It can be inferred that the decrease in CTR1 expression is a key factor contributing to the reduced copper clearance rate in the CSF (Liu et al., 2022). The divalent copper outside the cell is reduced to monovalent copper by the reducing enzyme 6-transmembrane epithelial antigen of prostate (STEAP) and then absorbed by the cell with CTR1. With the assistance of chaperone proteins such as the copper chaperone for superoxide dismutase (CCS) and superoxide dismutase 1 (SOD1), copper ions are transported to various subcellular organelles, including the mitochondria, the trans-Golgi network (TGN), and the nucleus. When the cytoplasmic copper level rises, ATP7A and ATP7B exit the TGN and facilitate copper excretion (Chen et al., 2022). Antioxidant-1 (ATOX1) is responsible for transporting copper to ATP7A and ATP7B within the TGN, delivering monovalent copper to newly synthesized metalloproteins or expelling it from the cell. Cytochrome c oxidase assembly protein 17 (COX17) is responsible for the transport of copper to the mitochondria in neuronal cells. Our recent study demonstrated that COX17 alterations are responsible for mitochondrial copper imbalance and AD-like pathology (Huang et al., 2024).

### 2.2 Contribution of copper metabolic dysregulation to the onset and development of AD

**Figure 1:**
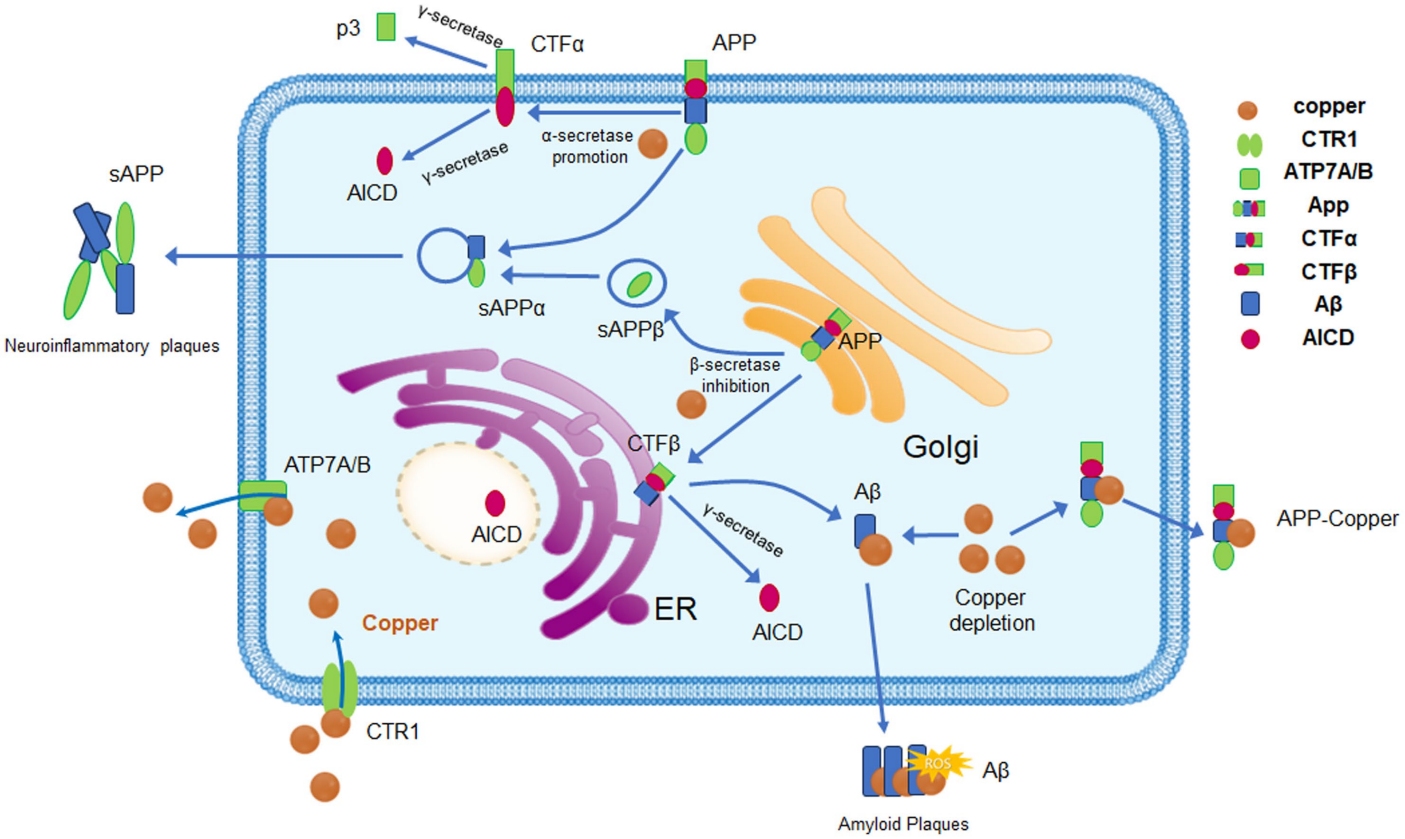
Copper homeostasis and its effects on APP processing. Copper enters cells via CTR1 and DMT1. When intracellular copper concentrations rise, the expression and activity of ATP7A and ATP7B are enhanced to promote copper excretion and maintain copper homeostasis. Copper can bind to specific regions of the APP, promoting the α-secretory pathway of APP, which affects the processing and metabolism of APP. After binding to APP, copper can be expelled from cells in the form of copper–APP complexes, a process that may lead to intracellular copper depletion and increased extracellular copper accumulation. The binding of copper to Aβ promotes the aggregation and precipitation of Aβ, forming the characteristic pathological feature of AD, amyloid plaques. Abnormal copper metabolism and Aβ deposition promote oxidative stress, trigger neuroinflammation, and exacerbate neuronal damage, which are significant influencing factors in the progression of AD.

Given the strict regulatory mechanisms of copper within the body, increased attention is paid to the relationship between copper dyshomeostasis and AD (Solioz, 2020). A meta-analysis of approximately 6,000 participants showed that copper levels were decreased in AD brain specimens, while copper and non-bound ceruloplasmin copper (non-Cp Cu) levels were increased in serum/plasma samples (Squitti et al., 2021; Squitti et al., 2023). The data indicated that the reduction of copper in the human frontal cortex exhibited brain-specific alterations, with a decrease in copper levels in the soluble extract and an increase in acid-extractable material (mainly containing insoluble plaques). This suggests that in the AD brain, there is a widespread disruption in the compartmentalization of copper (Figure 1) (Rembach et al., 2013). This study also suggests that the absorption of Aβ plaques may be responsible for copper dyshomeostasis. Moreover, the level of serum-free copper is associated with the severity of AD. Based on the levels of non-Cp Cu in serum/plasma samples, the AD patients can be subdivided into AD and CuAD. Between them, the CuAD patients showed higher levels of non-Cp Cu and three times worse performance in the cognition test (Squitti et al., 2023).

Using a perinatal copper deficiency mouse model, the research examined how neurons and glial cells react to a lack of copper from early life through to maturity, with increased activity in microglia and astrocytes, the appearance of vacuoles in cortical neurons, and the manifestation of neurological impairments in mice with copper deficiency (Noor et al., 2020). There are higher levels of soluble amyloid precursor protein extracellular domain (sAPPα) in their culture medium, whereas fibroblasts that have been genetically manipulated to be severely deficient in copper predominantly secrete sAPPβ and produce an increased amount of Aβ-cleaved APP intracellular domain (ACID). Copper deficiency also significantly reduces the steady-state levels of APP mRNA, while the protein levels of APP remain unchanged, suggesting that copper deficiency may hasten the translation of APP (Figure 1) (Summers et al., 2022).

Introducing the ATP7A gene into fibroblast cell lines results in a depletion of cellular copper and a reduction in APP expression, suggesting that copper deficiency caused by high ATP7A levels can inhibit the generation of Aβ by suppressing APP production (Noor et al., 2020). The brain metabolism of ATP7B-deficient mice becomes disrupted as early as 4 weeks after birth due to transient copper deficiency, which is subsequently repaired by the addition of copper. The transient copper deficiency within brain tissue correlates with an increase in ATP7A expression and a decrease in Ctr1 expression on the apical membrane of choroid plexus epithelial cells (Washington-Hughes et al., 2023).

### 2.3 Role of imbalance of intracellular and extracellular copper homeostasis in AD pathology

In AD, extracellular and intracellular copper has significant effects on the pathological process. An imbalance of extracellular copper ions promotes Aβ aggregation and interferes with Aβ clearance, while extracellular copper ions are capable of activating microglia, inducing their conversion to the proinflammatory (M1) type, releasing a variety of inflammatory mediators and exacerbating neuroinflammation. Intracellular copper ions cause oxidative stress and mitochondrial dysfunction through a variety of mechanisms. Intracellular copper ions have redox activity and can catalyze the Fenton reaction, which converts hydrogen peroxide (H₂O₂) to hydroxyl radicals (–OH), generating highly reactive oxygen species (ROS) that can damage cell membranes, proteins, and DNA. Hydroxyl radicals (–OH) can oxidize glutathione, converting it from reduced glutathione (GSH) to oxidized glutathione (GSSG), which reduces intracellular GSH levels, weakening cellular antioxidant defenses and leading to exacerbation of oxidative stress (Li et al., 2024). It has been shown that the main target of copper homeostatic imbalance leading to cellular oxidation–reduction disorders is in the mitochondria, where the production of reactive oxygen species damages the integrity of the mitochondrial membrane, leading to a decrease in the mitochondrial membrane potential and a decrease in the generation of ATP. In addition, copper ions can bind directly to lipoylated proteins in the tricarboxylic acid cycle, leading to the aggregation of lipoylated proteins and loss of Fe–S cluster containing proteins (Tsvetkov et al., 2022). This binding not only interferes with normal mitochondrial function but can also trigger a specific mode of cell death – copper death – leading to mitochondrial dysfunction and cell death.

Oxidative stress is a central pathological alteration in AD, and copper-binding proteins (e.g., albumin and ceruloplasmin) regulate the distribution and metabolism of copper in the body by binding copper ions, which these proteins can then transport to specific cells or organelles and reduce the oxidative activity of free copper ions. The N-terminal amino acid residues of albumin (e.g., Asp-Ala-His-Lys) can bind to copper ions, preventing them from catalyzing the production of ROS (Bar-Or et al., 2001). Ceruloplasmin can bind to copper in blood and tissue fluids and significantly inhibit copper-induced lipid peroxidation and may also play an indirect antioxidant role by promoting the synthesis of nitric oxide (NO) in the organism, as well as oxidizing Fe^2+^ to Fe^3+^ to promote the binding of iron to transferrin (Ganini et al., 2012; Halliwell, 2024). In addition, copper cyanoproteins may be indirectly involved in the metabolism of cholesterol and other lipids, further regulating the homeostasis of lipid metabolism and reducing the occurrence of lipid peroxidation. Thus, copper-binding proteins are important for maintaining redox balance in the extracellular environment and protecting cells from oxidative damage. These antioxidants may play a certain role in delaying the course of the disease, but there is still a long way to go in clinical trials, and the challenges of clinical application still need to be overcome, and future therapeutic strategies for antioxidants need to be further optimized.

## 3 Involvement of copper in amyloid plaque formation in AD

### 3.1 Cellular copper metabolism and the formation of Aβ deposits

Copper plays a significant role in AD progression, as evidenced by elevated copper levels in the blood of AD patients. This copper buildup impacts both the deposition and removal of Aβ. A 25% increase in copper content in senile plaques compared to the control group has been observed in AD mouse models (Bellingham et al., 2004). Another study has shown that mice exposed to copper through drinking water exhibited an increase in soluble amyloid protein Aβ1-42 in the brain (Wu et al., 2016).

The main pathological characteristic of AD is the deposition of amyloid proteins, with the primary structure of Aβ composed of 40–42 amino acid residues. During the process of amyloid protein generation, APP can produce amyloid-beta peptides through two pathways: the α-secretase and β-secretase pathways. α-Secretase cleaves APP in the plasma membrane or Golgi apparatus, resulting in the soluble N-terminal fragment of APP-α and the C-terminal fragment (CTFα). β-Secretase cleaves the precursor APP in the Golgi network and endosomes, generating the soluble N-terminal fragment of APPβ and the C-terminal fragment (CTFβ). Subsequently, CTFβ is cleaved again by γ-secretase to produce Aβ42 or Aβ40 and the intracellular domain of APP (AICD) (Spies et al., 2012). AICD is capable of relocating to the nucleus, where it functions as a transcription factor (Hung et al., 2013). In the cerebrospinal fluid of healthy individuals, Aβ1-40 is the most abundant Aβ fragment, with a concentration of 2–3 ng/mL, followed by the Aβ1-42 fragment, which has a concentration of 0.75 ng/mL. Aβ42 has the propensity to form oligomeric structures, which can induce neurotoxic effects and contribute to the development of amyloid plaques and neurofibrillary tangles. Notably, mutations in APP, presenilin (PS1), and presenilin (PS2) are responsible for familial AD and lead to an increased production of Aβ42. Mutations in the APP or PS1 gene can disrupt the equilibrium between Aβ42 synthesis and its elimination, potentially leading to the accumulation of Aβ42. Soluble Aβ oligomers within neuroinflammatory plaques may have a stronger toxic effect than Aβ42 in neurofibrillary tangles, and Aβ oligomers play significant roles in the onset and development of the early stages of AD (Figure 1) (Mroczko et al., 2018).

Recent studies suggest that the amyloid precursor protein (APP) plays a role in maintaining copper balance within the brain. In mice with the APP gene disrupted, there is an observed increase in brain copper levels, whereas excess APP expression results in a reduction of copper content in the brain (White et al., 1999). Copper interacts with the N-terminal domain of APP, which resides in the intracellular space. Upon reaching the plasma membrane, copper is liberated into the extracellular environment as part of a secreted APP–copper complex. This complex is then processed by secretases near the membrane, leading to its release. Meanwhile, the APP molecule devoid of copper is subjected to cleavage by intracellular secretases (Bayer et al., 2003). Strong evidence points to APP potentially functioning as a copper efflux protein in neurons, and its overexpression could be responsible for the observed copper deficiency in the brains of most AD transgenic mouse models (Lin et al., 2010). The overexpression of APP facilitates the transport of copper out of the cell, increasing copper efflux and leading to copper deficiency within the cell and a reduction in SOD-1 activity. Dietary copper stabilizes the activity of superoxide dismutase protein 1 in APP23 transgenic mice and reduces amyloid protein Aβ (Borchardt et al., 1999). On the other hand, APP has a gene expression level-dependent effect on the level of copper in neuronal cells, and its mechanism may be regulated by the N-terminal-binding domain of APP. APP and APLP2 are specifically involved in the regulation of copper balance in the liver and the cerebral cortex. Following the genetic deletion of these genes, there is a notable increase in copper concentrations in the cerebral cortex of approximately 40 and 16% for APP and APLP2 knockouts, respectively. However, certain studies indicate that the absence of APP and APLP2 expression, when knocked out individually, does not impact the levels of copper within neurons, suggesting that the two play a complementary role in neuronal copper homeostasis (Bellingham et al., 2004).

Using human fibroblasts with Menkes protein (MNK) deficiency, it has been established that a deficiency of copper notably decreases the levels of APP protein and suppresses the expression of the APP gene (Bellingham et al., 2004). Divalent copper binds to APP, promoting α-secretase cleavage and reducing β-secretase cleavage. Copper suppresses the amyloidogenic processing of APP and promotes the non-amyloidogenic pathway, thereby facilitating the cellular secretion of APP (Borchardt et al., 1999). Investigations into copper’s impact on APP dimerization and its metabolic processes, conducted through immunoprecipitation and immunostaining techniques, have revealed that copper enhances the dimerization of APP and boosts the overall production of Aβ. Concurrently, it decreases the ratio of Aβ42 to Aβ40 (Noda et al., 2013).

### 3.2 The copper-binding domain (CuBD) in APP

Copper ions can interact with APP through CuBD to regulate the production of Aβ. The CuBD exists in APP and is an N-terminal region located between residues 124–189 of APP, composed of an α-helix (residues 147–160) and three β-strands (residues 134–139, 163–174, and 177–188). It has been determined that residues His-147, His-151, Tyr-151, Tyr-168, and Met-170 are involved in coordinating copper (Singh et al., 2022). Research on the CuBD domain has revealed how it binds to copper ions. CuBD can strongly bind divalent copper *in vitro* and reduce it to monovalent copper, and the neurotoxicity of APP can be directly induced or enhanced by the oxidation of low-density lipoprotein through monovalent copper (White et al., 2002). APP can also act as a divalent copper reductase on the cell membrane, similar to Fre1 in yeast, providing monovalent copper that is subsequently taken up by copper transport proteins or delivered to receptor copper proteins. This, in turn, can shift the processing and secretion of APP to enhance the exclusion of copper (George et al., 2011). The CuBD within APP has been studied to reveal how it binds to copper ions. Research on the CuBD within APP has shed light on its copper-ion binding capabilities. This domain is capable of tightly binding to extracellular divalent copper ions and reducing them to monovalent form. The neurotoxic potential of APP can be either directly triggered or intensified by monovalent copper, particularly through the oxidation of low-density lipoproteins (George et al., 2011).

The binding of APP to copper can affect the interaction between Aβ and copper, leading to a reduction in reactive oxygen species (ROS) generated by Aβ–Cu complexes. Changes in copper levels can also influence the process of APP processing to produce Aβ, thereby controlling the generation of toxic Aβ (Kong et al., 2007). Interestingly, in the presence of the CuBD in the APP peptide, the absorption of divalent copper increases, protecting the damaged hippocampus and maintaining spatial memory capabilities. The APP–copper complex serves as a neuronal metal transporter or chaperone and its copper binding has been demonstrated to decrease Aβ levels. In the context of AD, the interaction between APP and divalent copper diminishes Aβ production by impeding the cleavage of APP.

### 3.3 The impact of copper binding to Aβ in AD

A growing body of research indicates that the buildup of Aβ aggregates, whether as oligomers or plaques, impairs synaptic function, triggers the development of neurofibrillary tangles, causes neuronal destruction, exacerbates impairments in learning and cognitive functions, and contributes to progressive dementia (Morris et al., 2014). The proteolytic processing of APP yields Aβ peptides, which are known for their neurotoxic properties. These peptides can self-assemble into soluble oligomeric structures and eventually condense into amyloid plaques (Bellingham et al., 2004). Amyloid plaques, predominantly made up of clumped Aβ proteins, are located in the brain’s extracellular spaces (Yan et al., 2009). The binding of copper to Aβ peptides catalyzes redox reactions, resulting in increased generation of oxidative free radicals, which accelerates the progression of AD (Huang et al., 2019). The role of metal ions in Aβ aggregation remains a topic of scientific debate. Aβ peptides typically exist in three aggregate forms: well-structured fibrillar aggregates, soluble oligomers, and large amorphous aggregates. Metal ions can enhance Aβ aggregation and accelerate the formation of amyloid fibrils or amorphous aggregates. Conversely, some studies suggest that divalent copper can reduce Aβ aggregation but leads to the formation of soluble, neurotoxic Aβ oligomers, thereby increasing neurotoxic effects (Rana and Sharma, 2019). Soluble oligomers of amyloid protein Aβ within neuroinflammatory plaques, rather than fibrillar Aβ42, may be the toxic agents acting in the initial phases of AD (Mroczko et al., 2018).

In transgenic models and post-mortem tissue sections of human AD brains, a lipid-rich ring encircles the core of Aβ amyloid plaques. Copper complexes with low-density lipoprotein in a dose-dependent manner, can lead to the formation of more complex structures and promote lipid peroxidation, especially in copper-rich environments. The potential co-localization of copper and lipids within Aβ plaques may serve as a basis for copper-induced oxidative stress and lipid precipitation (James et al., 2017). Oxidative stress leads to the formation of Aβ dimers with two covalently linked tyrosine residues, and this di-tyrosine cross-linking is considered a potential marker for the disease. The use of potent divalent copper chelating agents, such as the ATCUN peptide and l-histidine-l-alanine-l-histidine (HAH), effectively prevents the generation of reactive oxygen species induced by copper and the subsequent formation of di-tyrosine linked Aβ dimers (Di Natale et al., 2018).

### 3.4 Dysregulation of copper ions leads to abnormal tau phosphorylation and aggregation

Aβ deposition and tau hyperphosphorylation are two hallmarks of AD. Metal ion-induced deposition of Aβ and tau aggregation mediated by oxidative stress, neurotoxicity, synaptic plasticity, autophagy, and apoptosis have important effects on the development of AD. Environmental copper exposure can regulate tau phosphorylation through various mechanisms. One study found that exposure of 3xTg-A mice to 250 ppm copper for 3 or 9 months increased tau phosphorylation in hippocampal neurons, and abnormal Cdk5/p25 kinase activation induced the development of tau pathology (Kitazawa et al., 2009). Studies have shown that the phosphorylation level of tau protein at Ser396 is significantly increased after copper ion treatment in SH-SY5Y cells. On the one hand, copper ion directly binds to specific amino acid residues in tau protein, changing its conformation and making it more susceptible to phosphorylation (Voss et al., 2014). Copper induces a conformational change in the R3 peptide of the protein, which transforms it from a disordered state to a highly ordered α-helix structure and accelerates the formation of neurofibrillary tangles (NFTs) (Sensi et al., 2018). On the other hand, it indirectly affects tau phosphorylation by regulating kinase activity such as GSK-3β. In addition, copper ions can inhibit the activity of protein phosphatase 2A (PP2A) and reduce the dephosphorylation of tau protein, leading to an increase in its phosphorylation level (McKenzie-Nickson et al., 2018). Future research is still needed to explore the mechanism of the interaction between copper ions and tau protein in the pathogenesis of AD, develop therapeutic strategies for the disorder of copper ions, and provide new insights for the prevention and treatment of AD.

## 4 Role of copper in lipid metabolism in the development of AD

Cholesterol constitutes a significant portion of cell membrane structure and plays a role in hormone, bile acid, and vitamin production. Approximately 20% of the body’s cholesterol is found in the brain, including unesterified cholesterol, as well as small amounts of cholestanol and cholesterol esters. Metabolic defects in cholesterol can result in neurodegenerative diseases such as Huntington’s disease, Niemann-Pick disease type C, Parkinson’s disease, and AD (Zhang and Liu, 2015). A substantial amount of research indicates that alterations in cholesterol within lipid rafts are associated with the proteolysis of APP and the amyloidogenic process, which further leads to an increase in Aβ levels (Wang et al., 2021). A study involving 444 men, with a baseline age range of 70–89 years, has shown that men with elevated serum total cholesterol levels in middle age or the early elderly period face a heightened risk of AD in their later years. Elevated serum cholesterol is an independent risk factor for AD, and the association of apolipoprotein E (ApoE) alleles with AD might be mediated through mechanisms that involve elevated cholesterol levels (Notkola et al., 1998). Copper may exacerbate the burden of cholesterol in the brain. Studies have found that feeding rabbits a high-cholesterol diet in combination with copper exposure through drinking water can increase Aβ protein in the rabbit brain (Larry Sparks, 2004).

Cholesterol is involved in the development of the nervous system and the maintenance of physiological balance in the brain. In the adult nervous system, cholesterol cannot be obtained from peripheral sources. To meet the demand for cholesterol in the brain, it is primarily synthesized through *de novo* synthesis pathways. Cholesterol synthesis mainly relies on oligodendrocytes, especially astrocytes (Zhang and Liu, 2015). Research indicates that copper can affect the de novo synthesis pathway of cholesterol in astrocytes, leading to increased expression of the rate-limiting enzyme 3-hydroxy-3-methylglutaryl coenzyme A reductase (HMGCR) in cholesterol synthesis and promoting an increase in cholesterol synthesis. Cholesterol binds to the C-terminal domain of APP, promoting the release of Aβ and AICD. Aβ40 inhibits HMGCR, resulting in the accumulation of cholesterol.

Divalent copper boosts Aβ production, diminishes its degradation within brain tissue, and hinders Aβ clearance via the blood–brain barrier, leading to a higher accumulation of Aβ in the brain (Kim et al., 2013). Low-density lipoprotein receptor-related protein 1 (LRP1) primarily facilitates the transport of Aβ from the brain into the bloodstream. In experiments using mouse models, exposure to copper notably heightened neuroinflammation and reduced LRP1 levels, resulting in compromised Aβ clearance from brain tissue (Kitazawa et al., 2016). Lipophilic statin medications can more readily penetrate the blood–brain barrier and substantially lower circulating copper levels, demonstrating clinical benefits for the treatment of mild to moderate AD (Dai et al., 2021).

ApoE is a glycoprotein consisting of 299 amino acids and serves as the predominant apolipoprotein in humans, primarily facilitating lipid transport in plasma and cerebrospinal fluid. There are three main isoforms of ApoE in humans: ApoE2, ApoE3, and ApoE4, corresponding to the genetic variants ε2, ε3, and ε4. Notably, the ε4 variant of ApoE is recognized as a genetic risk factor for late-onset AD. ApoE plays a role in cholesterol transport within neurons and also aids in the movement of cholesterol from neurons to astrocytes. Studies have indicated that ApoE2 offers neuroprotective benefits by enhancing cholesterol efflux from both neurons and astrocytes. ApoE can alleviate copper-induced oxidative toxicity by forming a complex with copper. Studies have shown that the binding capacity of ApoE to copper is in the order of ApoE2 > ApoE3 > ApoE4, with ApoE4 having a weaker ability to bind copper, leading to impaired clearance of Aβ in the brain. In individuals with Wilson’s disease, the diminished copper binding affinity of ApoE4 leads to increased oxidative brain damage in those carrying the ApoE-ε4 genotype.

Lipid rafts are highly ordered micromembrane structures rich in cholesterol, sphingolipids, and saturated phospholipids. The β-secretase-mediated cleavage of APP predominantly takes place within lipid rafts (Yang et al., 2014). Aβ dimers, cellular prion protein (PrPc), and complexes of Aβ dimer with PrPc have been identified in lipid rafts. Concentrations of Fyn, the phosphorylated NR2B subunit of the NMDA receptor, glycogen synthase kinase 3, total tau, phosphorylated tau, and tau oligomers rise as these dimers build up in lipid rafts at the cellular and synaptic levels (Kawarabayashi et al., 2022; Hung et al., 2009). Cellular copper concentrations inversely relate to copper levels in lipid rafts, such that elevated intracellular copper leads to diminished processing of lipid rafts and reduced endocytosis of APP. In conditions of cellular copper deficiency in AD, the aggregation of Aβ and copper in lipid rafts promotes the formation of oxidatively active Aβ-divalent copper complexes, which in turn enhances the oxidation of cholesterol and lipids and the production of neurotoxic hydrogen peroxide. Copper regulates the binding of flotillin-2 to cholesterol-rich lipid raft domains; therefore, increasing cellular copper can reduce the binding of flotillin-2 to lipid rafts rich in cholesterol, thereby weakening the generation of Aβ (Hung et al., 2009).

## 5 Application of copper complexes and nanotechnology in the treatment and diagnosis of AD

### 5.1 The application of copper complexes in AD

Copper complexes are instrumental in the diagnosis and management of AD by sequestering copper within amyloid plaques (Ejaz et al., 2020). Chelating copper is supposed to decrease the formation of amyloid plaques and inhibit the ROS formation induced by the Cu–Aβ complex. Recently, a number of chelators have been developed and tested in cellular or animal models. Many of them have shown promising effects on decreasing Aβ aggregation and ROS production. Table 1 summarizes the most promising copper chelators in the treatment of AD.

**Table 1:** List of copper chelators studied with therapeutic potential for AD in recent years.

Parent compounds	Copper chelators	Binding ions	Testing models	Effects & targets	Reference (Author, Year)
Clioquinol	Clioquinol^*^	Cu, Zn	Neuroblastoma cell line, M1C cells; yeast cells	Decreases levels of phosphorylated, truncated, and oligomerized tau protein; reduces Aβ42 toxicity via the inhibition of oxidative damage	Lin et al. (2021)
8-hydroxyquinoline	PBT2 ^*^	Low Specificity Cu chelator	None	Ternary cu^2+^ complex with β-amyloid; interacts with the amyloid β 1–42 peptide	Drew (2023)
Pyridine derivatives	DPMGA	Cu, Zn	None	Remove Cu^2+^ from its amyloid-β complexes; inhibit ROS production	Jakusch et al. (2022)
Pyridine derivatives	Coumarin pyridine hybrids-3f	Cu, N/A	PC12 and SH-SY5Y cells	Anti-cholinesterase activity; reduction of ß-amyloid self and AChE-induced aggregation; against H_2_O_2_-induced cell death and amyloid toxicity	Babaei et al. (2022)
Pyridine derivatives	Bipyridine derivatives-4d	Cu	Aβ1–42 peptide injection SD rats; SH-SY5Y cells	Inhibition of Cu^2+^-induced Aβ1-42 aggregation; neuroprotective activity	Tan et al. (2024)
Pyridine derivatives	AS-HL1 and AS-HL2	Cu, N/A	None	Sequester Cu^2+^ from the Aβ − Cu complex; modulate the aggregation pathways of both metal-free and metal-bound amyloid-β	Rana et al. (2022)
	TDMQ20	A specific Cu chelator	5xFAD mice; TX mouse model (Willson diseases)	Inhibit *in vitro* the aerobic oxidation of ascorbate induced by cu-aβ1-16; reduce the oxidative stress in the mouse cortex; protect cholinergic system and synaptic transmission; decreased brain and increase the fecal excretion of copper; reduce memory impairments	Zhang et al. (2018), Zhao et al. (2021), Sun et al. (2022), Zhu et al. (2023)
Short peptides conjugated with a dual-functional fluorophoric amino acid (NGln).	P2N_Gln_	Cu, N/A	SH-SY5Y cells	P2N_Gln_ inhibits both Cu-dependent and -independent fibrillation of Aβ42, along with the subsequent toxicity induced by Aβ42; P2N_Gln_ exhibits inhibitory effects on the production of lipopolysaccharide (LPS)-induced ROS and reactive nitrogen species (RNS) in microglial cells	Mandal et al. (2024)
Dopamine Schiff base derivatives	SB-4	Cu, N/A	PC12 cell	SB-4 can capture Cu^2 +^ions in Aβ–Cu complexes, thereby inhibiting copper-induced Aβ aggregation and Aβ self-aggregation, as well as copper-catalyzed ROS production	Gharai et al. (2024)
8-hydroxyquinoline, pyridine or imidazole	Ruthenium-(II) Polypyridyl complexes	Cu, N/A	SH-SY5Y cells	Capture Cu^2+^ in Aβ and form a dimer; inhibit the production of ROS induced by Aβ; protect mitochondria from damage, and improve neuronal cell survival rate	Peng et al. (2021)
Thiosemicarbazone	Copper-bis-thiosemicarbazone-Complex	Cu, N/A	Ischemia model; familial AD derived brain endothelial-like cell (iBEC); C57BL6/J Mice	Copper delivery in the ischemic brains modulates the inflammatory response, specifically affecting the myeloid cells; CuII(atsm) also protects endogenous microglia against ischemic insult and reduces the proportion of invading monocytes; reduces the expression of proinflammatory cytokine genes but also reverses the detrimental effects of TNFα and IFNγ on the integrity and function of the AD iBEC monolayer; modifies the function at the blood–brain barrier	Huuskonen et al. (2017), Pyun et al. (2023)

*Compounds were tested with clinic trail; N/A, the specificity was not stated; None, no cell or animal model was used.

5-Chloro-7-iodo-8-hydroxyquinoline (CQ) and 5,7-dichloro-2[(diethylamino)methyl]-8-hydroxyquinoline (PBT2) were the two earliest studied compounds with therapeutic potential. Studies have found that CQ chelated approximately 83% of divalent copper from Aβ (1–42), while PBT2 chelated only about 59% of divalent copper from Aβ (1–42), indicating that CQ exhibits a greater binding affinity for divalent copper compared to PBT2 (Summers et al., 2022). Treatment with CQ has a dual effect on AD: it can inhibit metal-catalyzed Aβaggregation and toxicity by sequestering extracellular metals, and subsequently, by internalizing these bound metals into cells, it activates specific metalloprotein kinases, inducing the production of matrix metalloproteinases (MMPs), which in turn accelerates the degradation of Aβ (Squitti and Salustri, 2009).

PBT2 is a more effective Zn/Cu ionophore than CQ, capable of reducing the formation of hydrogen peroxide, has better blood–brain barrier permeability and higher solubility, and can also inhibit the accumulation of Aβ induced by copper and zinc *in vitro* (Ejaz et al., 2020). The metal chelator PBT2 is a tridentate ligand capable of forming binary and ternary copper complexes. PBT2 can interact with the highly stable but low-specificity copper–Aβ complexes and can form ternary copper–PBT2–NIM complexes with all NIM donor ligands (Drew, 2023). PBT2 is a Cu/Zn ionophore that can rapidly restore cognitive function in mouse models of AD. CQ and PBT2 were the only two copper chelators that have been tested in clinical trials. A pilot phase 2 clinical trial in patients with moderately severe AD showed that CQ decreased plasma Aβ-42 (Ritchie et al., 2003). However, CQ also showed neurotoxicity in animal experiments. So, further clinical trials were not carried out (Bareggi and Cornelli, 2012). A phase 2A double-blind, randomized, placebo-controlled trial found that a 250 mg dose of PBT2 was well-tolerated in AD patients over a 12-week treatment period, significantly reducing the levels of amyloid protein Aβ-42 in the cerebrospinal fluid (CSF) and improving executive function (Faux et al., 2010). It may be due to the poor specificity of PBT2, which can chelate both copper and zinc, affecting normal neural functions. Therefore, no larger-scale clinical trials have been conducted.

In recent years, a series of low-toxicity and highly specific copper chelators have been developed. These copper chelators have been tested in cellular and animal experiments and have shown potentially good therapeutic effects for AD (Table 1). Among them, TDMQ20 stands out as the most representative compound. TDMQ20 showed a higher specificity for copper chelation. Treatment of transgenic or non-transgenic AD animals with TDMQ20 showed decreased brain copper, reduced oxidative stress in the mouse cortex, protection of the cholinergic system and synaptic transmission, and reduced memory impairments.

Bis (thiosemicarbazone) complex with divalent copper, Cu^II^(atsm), as a promising metallodrug, has piqued interest in its potential for delivering copper to the brain, especially as a Cu-based radiopharmaceutical (Xiao et al., 2008). Treatment of APP-CHO cells with Cu^II^(atsm) derivatives and Zn^II^(atsm) complexes resulted in decreased levels of Aβ, indicating the potential of these compounds as therapeutic agents for AD. This involves the ability of Cu^II^(atsm) derivatives and Zn^II^(atsm) complexes to increase intracellular metal levels in Chinese hamster ovary cells overexpressing amyloid precursor protein (APP-CHO) and the subsequent impact on the extracellular levels of amyloid peptides (Donnelly et al., 2008). Cu^II^(atsm) may exert neurotherapeutic effects by alleviating neuroinflammation of the blood–brain barrier and the associated damage to the integrity of the blood–brain barrier (Wasielewska et al., 2024).

Cu^II^(atsm), which can cross the blood–brain barrier and biologically release ^64^Cu within the brain tissue, provides for real-time monitoring of copper distribution in the brain. ^64^Cu binds to amyloid plaques associated with AD. This complex has the potential to be used as a copper-64 radiopharmaceutical to assist in the positron emission computed tomography (PET) diagnosis of AD (Lim et al., 2010).

In the transgenic AD mice model, copper-64 radiopharmaceutical was used to assess brain copper transport in 6- to 8-month-old transgenic mice. Positron emission tomography (PET) image analysis indicated a significant increase in brain concentration of Cu-64 at 30 min and 24 h post-injection, and a faster clearance of Cu-64 from the brain in mice compared to the control group. Ex vivo autoradiography showed that the local brain distribution of Cu-64 at 24 h post-injection was consistent with the distribution of amyloid-β plaques in AD transgenic mice (Torres et al., 2016).

These results support the use of copper-64 radiopharmaceutical PET imaging as a clinical evaluation tool to study alterations in brain copper metabolism, which may have potential value in the diagnosis of AD. Currently, ^64^Cu-ATSM and its PET imaging technology are mainly used in the preclinical stage of AD diagnosis (Okazawa et al., 2022). More clinical trials are needed to ensure the safety, efficacy, and biodistribution characteristics of the tracer. Furthermore, the short half-life of copper-64 (~12.7 h) necessitates advanced radiopharmaceutical infrastructure for its production and distribution. This requirement poses significant challenges to its accessibility and scalability in clinical applications.

### 5.2 Application of nanotechnology in AD

Nanodrug delivery has great potential in the precise regulation of copper due to its precise targeting, controlled release, high loading capacity, and multi-drug delivery ability, and has made significant progress in the application of neurodegenerative diseases. Nanoparticles were designed to specifically bind copper(II) and efficiently capture and remove copper(II), thereby reducing its toxic effects on the brain (Cui et al., 2016). Prussian blue nanoparticles (PBNPs) have strong copper(II) chelation ability. The PB/RBC encapsulated on the cell membrane inhibits copper(II)-induced aggregation of Aβ monomers and eliminates the deposition of Aβ plaques, which can evade immune recognition *in vivo* and improve the anti-AD efficacy (Kowalczyk et al., 2021). PEGylated/CTAB silica nanoparticles showed a concentration-dependent drug-loading ability. The catechin content of PEGylated/CTAB silica nanoparticles could effectively regulate the toxicity of copper(II), alleviate the damage of nerve cells under oxidative stress conditions, and have a significant protective effect on nerve cells (Halevas et al., 2016). In the TASTPM transgenic mouse model, the polymeric nanoparticles carrying anti-Aβ antibody and ^64^Cu-GTSM enabled the efficient removal of Aβ plaques and PET imaging. Combined with animal models and clinical trials, further optimization of the formulation and preparation process of nanoparticles will provide more support for the diagnosis and treatment of AD (Le Droumaguet et al., 2012). Currently, the safety of nanotechnology in the diagnosis and treatment of AD needs to be further explored, especially the potential toxicity after long-term exposure, and more clinical trials are needed.

## 6 Conclusion

Copper plays an essential role in maintaining the physiological processes in the brain, and disruptions in its homeostasis are strongly implicated in the pathogenesis of AD. These pathological links include dysregulated Aβ aggregation, hyperphosphorylation of tau protein, oxidative stress, neuroinflammation, and mitochondrial dysfunction. Current mechanistic studies highlight the therapeutic potential of copper-targeted strategies, such as the use of copper chelators or modulators to reduce aberrant copper uptake and transport, offering a promising avenue for AD intervention. Nanocarriers further enhance therapeutic prospects by overcoming key pharmacological challenges. By encapsulating drugs, these systems enable efficient blood–brain barrier (BBB) penetration, improve drug stability and bioavailability, and minimize off-target distribution, thereby optimizing delivery to affected brain regions. However, critical gaps remain in both research and clinical translation. First, current methodologies for assessing cerebral copper imbalance remain limited, underscoring the urgent need to develop sensitive biomarkers for monitoring dynamic copper fluctuations in the brain. Second, the design of next-generation copper chelators with enhanced selectivity and specificity is imperative to maximize therapeutic efficacy while minimizing systemic side effects. While nanotechnology holds significant translational promise for copper-based AD therapies, its clinical application necessitates rigorous evaluation of long-term safety, biocompatibility, and sustained efficacy. Future efforts should prioritize interdisciplinary research to address these challenges and advance copper-targeted therapeutic paradigms.
